# Editorial: Deep learning methods and applications in brain imaging for the diagnosis of neurological and psychiatric disorders

**DOI:** 10.3389/fnins.2024.1497417

**Published:** 2024-10-01

**Authors:** Da Ma, Hao Zhang, Lei Wang

**Affiliations:** ^1^Department of Internal Medicine, Wake Forest University School of Medicine, Winston-Salem, NC, United States; ^2^School of Electronic Information, Central South University, Changsha, Hunan, China; ^3^Department of Psychiatry and Behavioral Health, Ohio State University Wexner Medical Center, Columbus, OH, United States

**Keywords:** deep learning, artificial intelligence, brain imaging, neuroimaging, neurological disorder, psychiatric disorder

## Introduction

Neuroimaging-based biomarkers have been used extensively for various neurological and psychiatric disorders, although accurate brain image-based diagnosis at the individual level remains elusive (Masdeu, 2011; Sui et al., 2020). In recent years, deep learning techniques have achieved remarkable success in fields such as computer vision and natural language processing, given their ability to learn complex patterns from large amounts of data (Zhang et al., 2020; Quaak et al., 2021). Applying deep learning to neuroimaging-assisted diagnosis, while promising, face challenges such as insufficiently labeled data, difficulty in interpretation, data heterogeneity, and multi-modal integration (Yan et al., 2022). This Research Topic highlights the development and application of cutting-edge deep learning research using neuroimaging for brain disorders, marking a collective effort to address these challenges.

The topics of the studies include differential diagnoses for brain tumors (Chen et al.; Zhang et al.) and dementia (Ma et al.) subtypes, early detection (Lang et al.; Huang et al.; Chattopadhyay et al.; Nie et al.; Liu et al.), and intervention (Yu and Fang) for neurological and neuropsychiatric disorders, as well as intracranial fluid segmentation (Puzio et al.). Various neuroimaging modalities were utilized, including structural magnetic resonance imaging (MRI), diffusion tensor imaging (DTI), functional MRI (fMRI), Electroencephalogram (EEG), and computerized tomography (CT). A diverse range of advanced deep neural network architectures were developed and evaluated, including convolutional and graph neural networks (CNN, GNN), multi-modal neuroimaging feature fusion, vision transformers, and composited architectures.

### Differential diagnosis, prognosis, and treatment response evaluation

Distinguishing different tumor types is fundamental for precision cancer treatment (Shoeibi et al., 2023; Wen et al., 2023). Chen et al. performed effective feature extraction of T1-weighted MRI by fusing multiple CNN models through pairwise feature summation, achieving an accurate classification performance of over 0.97. Zhang et al. employed a hybrid approach using EfficientNet-based feature extraction followed by a support vector machine (SVM), demonstrating comparable performance and identifying tumor regions with a Grad-CAM-based saliency map.

Identifying dementia subtypes is also crucial for personalized medicine for neurodegeneration (Ma et al., 2020; Chouliaras and O'Brien, 2023; Haller et al., 2023; Wen et al., 2023). Ma et al. introduced a multi-level, multi-type feature embedding and fusion approach to differentiate three heterogeneity clinical phenotypes of FTD: behavioral-variant (bvFTD), semantic-variant primary progressive aphasia (svPPA), and nonfluent-variant-PPA (nfvPPA), achieving a balanced accuracy of 0.84. The integrated-gradient-based explainable AI approach demonstrated more localized differential subtype patterns than groupwise statistical mapping.

Excessive accumulation of β-amyloid in the brain, a hallmark of Alzheimer's disease (AD) can be detected using PET (Jack et al., 2010; Tosun et al., 2021). Chattopadhyay et al. evaluated various machine-learning approaches to achieve this, including: (1) feature-engineered approaches, including logistic regression, XGBoost, and shallow artificial neural networks (ANN), (2) deep learning models with 2D/3D convolutional neural networks (CNN), (3) hybrid ANN-CNN models, (4) transfer learning on pretrained CNNs, and (5) Vision Transformers (MINiT). Validating a large-scale MRI/PET-paired dataset from 1,847 elderly participants, the hybrid ANN-CNN and 3D vision transformer achieved the best performance, reaching a balanced accuracy and an F1 score of around 0.8.

For neuropsychiatric disorder, Yu and Fang examined the effectiveness of exercise in Attention Deficit Hyperactivity Disorder (ADHD) patients by predicting diagnosis and intervention response through a composited approach. Random Forest was first used to select features from multi-source data. A Time Convolutional Network (TCN) was then applied to capture the behavioral and physiological signals related to motor activities over time. An Adaptive Control of Thought-Rational (ACT-R) model was used to simulate ADHD patients' cognitive processes, behavioral responses, and symptoms. Evaluation of multiple datasets demonstrated generalizable performance.

### Brain network and EEG analysis

GNN has shown promising capability to analyze whole-brain connectivity to gain insight of neuropsychiatric disorders (Bessadok et al., 2023). Brain networks can be derived either from functional connectivity or structural connectivity derived from fMRI and DTI accordingly. Lang et al. introduced a novel GNN approach incorporating task-specific prior (TSP) knowledge to improve the characterization of the functional connectome patterns, demonstrating state-of-the-art performance in classifying different neuropsychiatric disorders, including ADHD, autism, and schizophrenia, as well as distinct task-specific connectivity patterns for various neuropsychiatric disorders. Huang et al. introduced a novel multi-layer brain network graph embedding to integrate multi-modal data. Complementary and unique information from structural and functional connectivity was captured through traversing nodes in each layer, with group differences computed at both the nodal and network levels, improving schizophrenia and bipolar disorder classification.

Nie et al. introduced a composited deep learning model on the electroencephalogram (EEG) data to capture the brain's electrophysiological signals for the early diagnosis of epilepsy. Fast Fourier Transform (FFT) extracted EEG signals were fed into a nested CNN-LSTM model, demonstrating state-of-the-art performance (accuracy/sensitivity/specificity = 0.96/0.93/0.96), exceeding state-of-the-art methods. Liu et al. introduced an attention-based multi-semantic dynamic graph convolutional network (AMD-GCN) to detect fatigue from EEG functional connectivity data. AMD-GCN integrates multiple modules, including channel-attention to assign weights to different input features, a multi-semantic dynamic graph convolution to capture node dependency, and a spatial-attention mechanism to remove redundant spatial node information, achieving the best classification performance on the SEED-VIG public dataset (0.90 accuracy) on fatigue detection.

### Intracranial fluid segmentation in emergency settings

Image segmentation is a crucial step in clinical assessment of brain disease (Siddique et al., 2021). Puzio et al. conducted intracranial compartment (ICC) and cerebrospinal fluid (CSF) segmentation on emergency trauma head CT scans for triaging high-risk patients with traumatic brain injury for further neurosurgical treatment, achieving a dice similarity score of 0.765/0.567/0.574/556 for ICC, right/left supratentorial and infratentorial CSF regions. Comparison between automated and manual segmentation on CSF compartments demonstrated high inter-class correlation. The ICC to CSF ratio demonstrated clinical relevance in identifying patients who require surgical intervention.

## Conclusions and discussions

This Research Topic presented a collection of the latest advancements in deep learning techniques on neuroimaging, demonstrating the effectiveness in diagnosing brain disorders such as neurodegeneration, neuropsychiatric symptoms, brain tumors, and traumatic brain injury. Despite these successes, challenges remain to be addressed to facilitate further clinical translation in biomedical and health applications. First, comprehensive evaluations on standard and diverse datasets will be critical for benchmarking model performance, ensuring generalizability and translatability. Second, beyond integrating multi-modal neuroimaging data, future studies would incorporate multidimensional data such as non-imaging biomarkers and electronic health records (EHR). Finally, more advanced explainable AI approaches, such as counterfactual analysis to infer causal relationships and uncertainty measurements, are needed to ensure trustworthiness, human-in-the-loop, and successful adoption of AI models.

## References

[B1] Bessadok A. Mahjoub M. A. Rekik I. (2023). Graph neural networks in network neuroscience. IEEE Trans. Pattern Anal. Mach. Intell. 45, 5833–5848. 10.1109/TPAMI.2022.3209686 36155474

[B2] Chouliaras L. O'Brien J. T. (2023). The use of neuroimaging techniques in the early and differential diagnosis of dementia. Mol. Psychiatry 28, 4084–4097. 10.1038/s41380-023-02215-8 37608222 PMC10827668

[B3] Haller S. Jäger H. R. Vernooij M. W. Barkhof F. (2023). Neuroimaging in dementia: more than typical Alzheimer disease. Radiology 308:e230173. 10.1148/radiol.230173 37724973

[B4] Jack C. R. Wiste H. J. Vemuri P. Weigand S. D. Senjem M. L. Zeng G.. (2010). Brain beta-amyloid measures and magnetic resonance imaging atrophy both predict time-to-progression from mild cognitive impairment to Alzheimer's disease. Brain 133, 3336–3348. 10.1093/brain/awq277 20935035 PMC2965425

[B5] Ma D. Lu D. Popuri K. Wang L. Beg M. F. Alzheimer's Disease Neuroimaging Initiative (2020). Differential diagnosis of frontotemporal dementia, Alzheimer's disease, and normal aging using a multi-scale multi-type feature generative adversarial deep neural network on structural magnetic resonance images. Front. Neurosci. 14:853. 10.3389/fnins.2020.00853 33192235 PMC7643018

[B6] Masdeu J. C. (2011). Neuroimaging in psychiatric disorders. Neurother. J. Am. Soc. Exp. Neurother. 8, 93–102. 10.1007/s13311-010-0006-0 21274689 PMC3052989

[B7] Quaak M. van de Mortel L. Thomas R. M. van Wingen G. (2021). Deep learning applications for the classification of psychiatric disorders using neuroimaging data: systematic review and meta-analysis. NeuroImage Clin. 30:102584. 10.1016/j.nicl.2021.102584 33677240 PMC8209481

[B8] Shoeibi A. Khodatars M. Jafari M. Ghassemi N. Moridian P. Alizadehsani R.. (2023). Diagnosis of brain diseases in fusion of neuroimaging modalities using deep learning: a review. Inf. Fusion 93, 85–117. 10.1016/j.inffus.2022.12.010

[B9] Siddique N. Paheding S. Elkin C. P. Devabhaktuni V. (2021). U-net and its variants for medical image segmentation: a review of theory and applications. IEEE Access 9, 82031–82057. 10.1109/ACCESS.2021.3086020

[B10] Sui J. Jiang R. Bustillo J. Calhoun V. (2020). Neuroimaging-based individualized prediction of cognition and behavior for mental disorders and health: methods and promises. Biol. Psychiatry 88, 818–828. 10.1016/j.biopsych.2020.02.016 32336400 PMC7483317

[B11] Tosun D. Veitch D. Aisen P Jack C. R. Jr. Jagust W. J. Petersen R. C.. (2021). Detection of β-amyloid positivity in Alzheimer's disease neuroimaging initiative participants with demographics, cognition, MRI and plasma biomarkers. Brain Commun. 3:fcab008. 10.1093/braincomms/fcab008 33842885 PMC8023542

[B12] Wen J. Varol E. Yang Z. Hwang G. Dwyer D. Kazerooni A. F.. (2023). “Subtyping brain diseases from imaging data,” in Machine Learning for Brain Disorders, ed. O. Colliot (New York, NY: Humana). Available at: http://www.ncbi.nlm.nih.gov/books/NBK597476/ (accessed September 15, 2024). 37988509

[B13] Yan W. Qu G. Hu W. Abrol A. Cai B. Qiao C.. (2022). Deep learning in neuroimaging: promises and challenges. IEEE Signal Process. Mag. 39, 87–98. 10.1109/MSP.2021.3128348

[B14] Zhang L. Wang M. Liu M. Zhang D. (2020). A survey on deep learning for neuroimaging-based brain disorder analysis. Front. Neurosci. 14:779. 10.3389/fnins.2020.00779 33117114 PMC7578242

